# DSC Phase Transition Profiles Analyzed by Control Charts to Determine Markers for the Authenticity and Deterioration of Flaxseed Oil during Storage

**DOI:** 10.3390/foods12152954

**Published:** 2023-08-04

**Authors:** Mahbuba Islam, Anna Kaczmarek, Anna Grygier, Jolanta Tomaszewska-Gras

**Affiliations:** 1 Department of Food Quality and Safety Management, Poznań University of Life Sciences, ul. Wojska Polskiego 31/33, 60-624 Poznań, Poland; mahbuba.islam@up.poznan.pl (M.I.); anna.kaczmarek@up.poznan.pl (A.K.); 2 Department of Food Technology of Plant Origin, Poznań University of Life Sciences, ul. Wojska Polskiego 31/33, 60-624 Poznań, Poland; anna.grygier@up.poznan.pl

**Keywords:** differential scanning calorimetry, melting profile, storage analysis, plant oils, stability, authenticity

## Abstract

An approach of implementing X-bar and R control charts as a statistical control tool to monitor the changes in the melting profile of fresh and stored flaxseed oils by differential scanning calorimetry (DSC) was used. Phase transition melting profiles were collected after 0, 2, 4, and 6 months of storing flaxseed oils, originating from five different cultivars. Four peaks at around −36, −30, −25, and −12 °C were identified using the deconvolution analysis procedure, which enabled the data to be collected at peak temperature (T), peak height (h), the peak area (A), and the percentages of the area (P A), as well as the ratio calculated from these parameters. Control charts obtained for the second peak of the melting profile showed a significant decrease of peak height (h2) from 0.50 to 0.39 W/g and the percentage of the area (P A2) from 50 to 38%, within the storage time (*p* ≤ 0.05); thus, they were considered to be indicators of oil deterioration. Strong negative correlations of the unstable parameters of DSC with chemical indicators of the oils’ oxidative stability (PV, p-AV, TOTOX) were found. For DSC parameters, related to the first peak (h1, A1) and the third peak (h3, A3), changes were statistically not significant within storage (*p* > 0.05); thus, they can be used as markers of flaxseed oil authenticity. The study demonstrated that X-bar and R control charts could effectively monitor changes in the specific peaks and calculated ratios from the DSC melting profile of fresh and stored flaxseed oils, serving as reliable indicators of oil deterioration.

## 1. Introduction

One of the most popular cold-pressed plant oils, found on the shelf of almost every supermarket, is flaxseed oil. The unique profile of fatty acids ranked this oil among the healthiest edible oils in terms of the content of polyunsaturated fatty acids (PUFA), especially from the n-3 (omega-3) group. Also called linseed, this multipurpose plant belongs to the Linaceae family and grows well in the temperate climate zone. According to FAOSTAT’s report from 2020, annually, 738,940 thousand tons of flaxseed oil are produced to meet the increasing demand, with China dominating the production, followed by Belgium, the United States, Germany, and India [1]. Research has demonstrated that the quality and utility of the products obtained from this plant depends on the variety [2], climate [3], seed maturity [4], and extraction process [5]. Reasonably, edible flaxseed oil is formulated using the cold-pressing process, which enables the retention of an outstanding combination of triacylglycerols (TAG’s) and acylglycerols (mono- and diacylglycerol), and other bioactive compounds (i.e., phenols, sterols, tocopherols, phospholipids, lignans, and pigments). Thus, the cold pressing of seeds has become popular nowadays, and some of these valuable compounds, e.g., tocopherols and phenols, play a role in the stability of the oils and exert significant antioxidant properties upon consumption. Similar to *Lallemantia iberica*, [6] cold-pressed flaxseed oil has been acknowledged as one of the richest in polyunsaturated fatty acids (PUFA’s), especially α-linolenic fatty acid (ALA; C18:3, n-3) and linoleic acid (LA; C18:2, n-6), amounting to 50–63% and 16–26%, respectively [2,7], which are crucial for the human diet. Accompanied to the fatty acids, the presence of fat-soluble vitamins such as vitamin E (mainly as γ-tocopherol), phyto-estrogenic lignans (secoisolariciresinol diglucoside; SDG), and dietary fiber has made this non-traditional oil very popular amongst researchers, due to its manifold applications [8,9,10]. Here it is worth noting that flaxseed oils are used not only as edible oils in bottles, but also in pharmacies as a carrier of fat-soluble supplements (e.g., vitamin D). Meanwhile, the benefits of cold-pressed flaxseed oil come with an inevitable oddity, since the presence of many PUFAs make this oil very prone to peroxidation, which can additionally be accelerated by other natural compounds present in the oil, such as chlorophylls or phospholipids. Thus, ongoing anti- and pro-oxidant interactions makes cold-pressed oils less predictable in terms of their shelf-life stability [11]. Although the regular shelf life for vegetable oils has been accepted as 6 to 12 months [11], instructions from flaxseed oil producers clearly state the oxidation proneness of this oil, as the stability time has been pinned down at 5 weeks to 3 months [12]. This uncertain shelf-life assumption urged researchers to find the reasons and indicators responsible for the deterioration of flaxseed oil quality. In chemical analysis, alongside p-anisidine (p-AV), acid (AV) and peroxide (PV) values are the most common indicators for observing the stage of rancidity. However, other methods were also used, for instance, conjugated diene (CD) and triene (CT) values [13,14], a Rancimat analysis [15], and an isothermal and dynamic analysis by DSC [14].

With the furtherance of achievements in food science, the ultimate strategy nowadays is to use instruments, which are supposed to be not only ecological and environmentally friendly, but also a time-saver. Recent advancements in thermo-analytical assessments have contributed substantially to the analysis of fats and oils characterization. Differential scanning calorimetry (DSC) is an advanced analytical instrument which has broad applications dedicated to explaining the thermal behavior of lipid compounds, and is thus able to provide significant data regarding the authenticity and stability profiles of fats and oils [16]. Several research studies have proven the effective use of the DSC technique to explore the oxidative deterioration resulting from normal or accelerated shelf-life tests [17,18,19]. Compared to conventional chemical analyses, an assessment by DSC offers a significant database, expressed as the exothermic/endothermic phase transition phenomena manifested by curve changes as a function of temperature, which is related to the lipids composition of the oil. Thus, the unique and specific DSC curves for flaxseed oils can also be obtained as the cooling and melting profile [2,20]. Bearing in mind that such a crystallization or melting profile could be used as a fingerprint to assess authenticity and to ensure the safety and quality of this oil, it is important to investigate whether these profiles are stable over the shelf life of the oils. So far, there have been no such studies showing how the crystallization or melting profile changes within the storage time. Considering that flaxseed oil may be nutritionally degraded due to adulteration with other less expensive oils or due to rancidity, it is important to monitor for both of these risks. Thus, in this study, the aim was to evaluate the changes in the DSC melting profiles of flaxseed oil of various cultivars over their shelf life using a control chart as one of the statistical process control tools. The novelty of this study lies in its distinctive approach of employing X-bar and R control charts to monitor the changes in the DSC melting profile of both fresh and stored flaxseed oils over a period of six months. This approach enables the identification of reliable indicators for oil deterioration and authenticity, utilizing specific peaks and calculated ratios. Moreover, this rapid and environmentally friendly method for assessing the oil quality and stability holds significant importance, especially for flaxseed oil, which is highly prone to oxidation during extended storage. To the best of the authors’ knowledge, this particular approach has not been previously presented in any other study.

## 2. Materials

### 2.1. Materials

For the experiment, 15 kg of seeds of each cultivar, i.e., Bukoz (FL BU), Dolguniec (FL DL), Szafir (FL SZA, FL SZB) and of an unknown variety (FL NN) of flax were cold pressed to obtain the oils. All seeds were pressed in the SEMCO manufactory to obtain the oils at the same conditions by keeping the temperature below 50 °C. The pressed oils were left for 24 h for decantation and stored in brown glass bottles, which were used to store the oils as they offer excellent protection against light exposure, particularly harmful UV rays, and also to replicate the similar conditions as they are available in the market. A storage analysis was carried out for the samples from the fresh condition until the sixth month of shelf life. For every period of storage (0, 2nd, 4th, and 6th month) freshly opened bottles of flaxseed oil samples were used in order to perform all the analyses. During the shelf life, the samples were kept air-tight at room temperature (23–25 °C) by the window, where they were exposed to ambient natural sunlight with the aim to simulate real-life conditions that the oils may encounter during transportation, distribution, or in consumer households.

### 2.2. Methods

#### 2.2.1. Fatty Acid Composition

A Gas Chromatography-Flame Ionization Detector (GC-FID) was employed for the determination of fatty acid composition. An amount of 15 mg of oil was dissolved in 1 mL of hexane (for HPLC, Sigma Aldrich, St. Louis, MO, USA), and 1 mL of 0.4 N sodium methoxide was added. The samples were stirred and left for 15 min, then 5 mL of distilled water was added, and the top layer was taken off. By following the AOCS official method [21], fatty acid methyl esters were analyzed using a Trace 1300 chromatograph (Thermo Fisher Scientific, Waltham, MA, USA). Separation was performed on a Supelcowax 10 capillary column (30 m × 0.2 mm × 0.2 μm), and an injection was performed in the splitless mode. The sample volume was 1 µL. Hydrogen was used as the carrier gas. The initial oven temperature was 160 °C and was increased to 220 °C with a rate of 12 °C. The temperature of 220 °C was maintained for 20 min. The quantification of fatty acids was performed using the percentage of the area method, where individual fatty acids were measured based on their retention times via a comparison with the standard of 37-Component FAME Mix (Supelco, Sigma Aldrich). All the samples were analyzed in two replications.

#### 2.2.2. Chemical Analyses of the Oxidative Stability of Fresh and Stored Flaxseed Oil

The acid value (AV) was determined by using a volumetric titration formula following the standard AOCS method [22]. The peroxide value (PV) was measured by determining the milliequivalents of excess active oxygen content in the oil samples by following the standard ISO method [23]. The secondary oxidation products were determined by measuring the p-Anisidine values (p-AV) according to the standard [24]. The total oxidation rate of the oil samples was expressed as a TOTOX value and calculated from the formula based on the reported p-Anisidine and peroxide value data, TOTOX = p-AV + 2PV [23].

#### 2.2.3. Melting Phase Transition Analysis by Differential Scanning Calorimetry (DSC) of Flaxseed Oil during Storage

A melting analysis of flaxseed oils was carried out with modifications according to the method used for butterfat [25]. A PerkinElmer differential scanning calorimeter (DSC 8500 PerkinElmer, Waltham, MA, USA), equipped with an Intracooler II and running with Pyris software (PerkinElmer, Waltham, MA, USA), was used. Samples of ca. 6–7 mg were weighed into aluminum pans of 20 µL (PerkinElmer, No. 0219-0062, Waltham, MA, USA) and hermetically sealed. The reference was an empty, hermetically sealed aluminum pan, and nitrogen (99.999% purity) was used as the purge gas. The analysis started with cooling the oil sample at a scanning rate of 2 °C/min from a temperature of 30 °C to −65 °C, after which it was heated at scanning rates of 5 °C/min from −65 °C to 30 °C. For each measurement at a given scanning rate, the calibration procedure was completed with the correct scanning rate. After the analysis, the DSC files were converted into the ASCII format and were analyzed using Origin Pro software, version 2020 (OriginLab Corporation, Northampton, MA, USA). The temperature of each peak (T), the amplitude of each peak height (h), and area of each peak of the transition (A) were determined from the curves via the fitting procedures. The multicomponent DSC curves were deconvoluted with PeakFit, using the nonlinear least squares fitting procedure included in the Origin Pro software. All measurements were performed in duplicate for each sample.

#### 2.2.4. Statistical Analysis

The results were presented in the form of mean and standard deviation. A statistical analysis of the recorded results was performed using Statistica 13.3 software (TIBCO Software Inc., Palo Alto, CA, USA) at a significance level of α = 0.05. The first stage in the statistical analysis consisted of verifying the variance homogeneity using the Hartley–Cochran–Bartlett test. In the case of variance homogeneity, a one-way analysis of the variance (ANOVA) was used, and Tukey’s test was applied to create statistically homogeneous groups. In turn, when the variances were not homogeneous, non-parametric tests were used, i.e., ANOVA and the Kruskal–Wallis rank test. Additionally, a principal component analysis (PCA) was performed to show the relationships between the variables and detect some patterns between the variables and the objects. X-bar and R (arithmetic mean and range) control charts were used to test the stability of selected melting profile parameters. The X-bar and R control chart were used with continuous/variable data when a subgroup or sample size was between 2 and 15. The X-bar chart is based on a calculation of the average level of the parameter being monitored, while the R chart showed the range, i.e., the difference between the smallest and the largest value in each sample at each time point of storage, revealing the variability of the variation.

## 3. Results

### 3.1. Physicochemical Characteristics of Fresh and Stored Flaxseed Oils

Physicochemical characteristics of fresh (0 month) and stored (6th month) flaxseed oils included the fatty acid composition and chemical analyses of the oxidative stability (AV, PV, p-AV, and TOTOX).

#### 3.1.1. Fatty Acid Content

**Figure 1:**
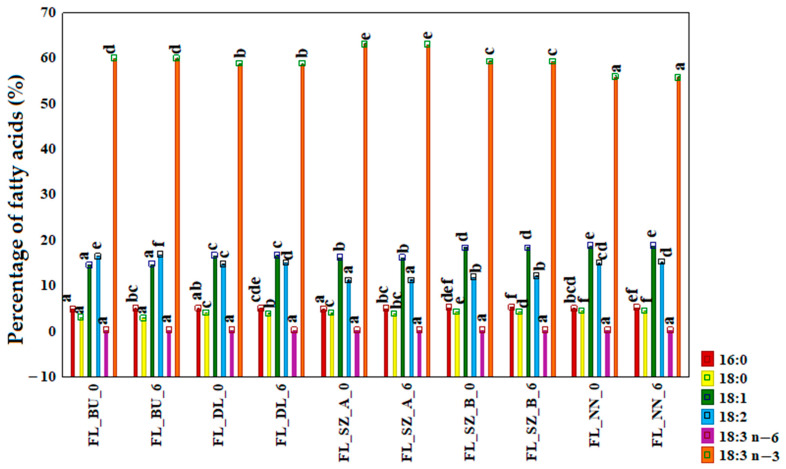
Fatty acids composition expressed as a percentage of total fatty acids (%) for fresh (0 month) and stored (6th month) flaxseed oils. a–f—different superscript letters above bars indicate significant differences analyzed for each fatty acid (*p* ≤ 0.05). FL_BU_0 and FL_BU_6 (Bukoz variety at 0 month and after 6 months, analogously), FL_DL (Dolguniec variety), FL_SZA, FL_SZB (Szafir variety), and FL NN (Unknown variety).

The abundance of PUFA (up to 73%) with 16% of monounsaturated fatty acids (MUFA) and 8% of saturated fatty acids (SFA) was determined in all flaxseed oils, as shown in Figure 1. The predominant fatty acid was α-linolenic acid (ALA, C18:3, n-3), which contributed up to 63% of the total fatty acids. Between the oil samples, FL_NN showed the lowest quantity of ALA (55%), of which FL_SZA showed the highest quantity of 63%. This quantity is comparatively higher than that found in other studies on commercial oils from the same region [15]. Nevertheless, the value aligns with the data reported for high-performing linseed cultivars. For instance, Florinda, Lirina, and Floriana cultivars, monitored over nine years, exhibited ALA levels accounting for 63.04%, 63.25%, and even 64.10%, respectively [26]. Despite the presence of three unsaturated carbon bonds, this fatty acid remained stable and showed no significant differences (*p* > 0.05) for all the oil samples after six months of storage. Next, the abundant PUFA, linoleic acid (C18:2, LA), was detected as the highest at 16% for the Bukoz variety. The data obtained are comparable to the results of other researchers [27]. Of the MUFAs, oleic acid is distributed amongst the varieties in a range from 14% to 18% of the total FA and no significant differences were observed between 0 and 6 months of storage for all the oil samples (*p* > 0.05). Concerning the presence of the two SFAs, i.e., palmitic acid (C16:0) and stearic acid (C18:0), up to 4% and 3%, respectively, was observed. Summing up the entire dataset for 0 and 6 months of oil storage showed that there were no significant differences in the composition of fatty acids between fresh and stored oils (*p* > 0.05). This finding about the changes in fatty acids after the storage time analysis can be compared with another study on flaxseeds stored for 128 days, which found no changes in fatty acid composition after the storage time [28]. Also, another study showed that, cold-pressed flaxseed oils stored for six months at room temperature exhibited no significant differences for SFA, MUFA or PUFA, which is linear to the data found from our study [29]. 

#### 3.1.2. Oxidative Stability Analysis of Flaxseed Oil

A conventional chemical analysis was carried out to detect any deterioration of the oil samples after six months of shelf life. The acid value (AV) measurement results, expressing the amount of free fatty acids, are shown in Table 1. The analyzed samples from the different varieties showed increased hydrolytic phenomena after six months, as the values increased for all of them. Particularly, the highest AV was found for the sample FL_NN, which was 3.25 (statistically significant at *p* ≤ 0.05) after shelf life. However, none of the samples exceeded the standard limit (4.0 mg KOH/g oil) provided by the Codex Alimentarius [30]. A similar propensity was assessed for the peroxide values and p-Anisidine values obtained for all varieties. The peroxide values measured the primary oxidation compound formation in samples like aldehydes and ketones, which gradually generates a musty smell and promotes rancidity. After shelf life, the FL_SZB sample produced the highest (*p* ≤ 0.05) level of the primary oxidation product (15.57), which is still within the range of the acceptable limit from the given standard (up to 15 milliequivalents of active oxygen/kg oil) [30]. For the storage time analysis, the p-Anisidine values are considered more reliable, as they provide information about secondary oxidation products (e.g., 2-alkenals and 2,4-alkadienals as a result of hydroperoxide decomposition, and unsaturated aldehydes) [31]. As an indicator of the oxidative rancidity, the p-AV values presented in Table 1 show that all samples presented an increased level of secondary oxidation phenomena, where the FL_NN sample was the highest at 2.47 (*p* ≤ 0.05). The overall oxidation state can be assessed by means of the TOTOX value, where the sample FL_SZB exhibits the highest level of oxidation (33.61) amongst all varieties after the shelf-life period. Changes in the oxidation states determined by other authors can be compared with this study. Similar results were found by authors who stored flaxseed oils at room temperature and presented a significant rise in the oxidative status determined by AV, PV, and p-AV values [13]. Authors from the same region [15] performed a storage analysis of the commercial oils after storing them in a refrigerator (4 ± 2 °C). Their results showed a significant level of increase for all AV, PV, and p-AV values after six months of shelf life.

The unexpected results showed no changes in the percentage of individual fatty acids after 6 months of storage (Figure 1), and significant changes in the oxidative stability indicators (Table 1) may suggest that the oxidative changes that occur in the oils come from the fraction of free fatty acids or mono- or diacylglycerols. It should be noted here that the procedure for determining the fatty acids includes extraction with hexane, which dissolves only the TAG, unlike the free fatty acids and the rest of the acylglycerols.

### 3.2. Analysis of the DSC Melting Profiles of Flaxseed Oils during Shelf Life

#### DSC Melting Profile Changes during Storage of Flaxseed Oil

**Figure 2:**
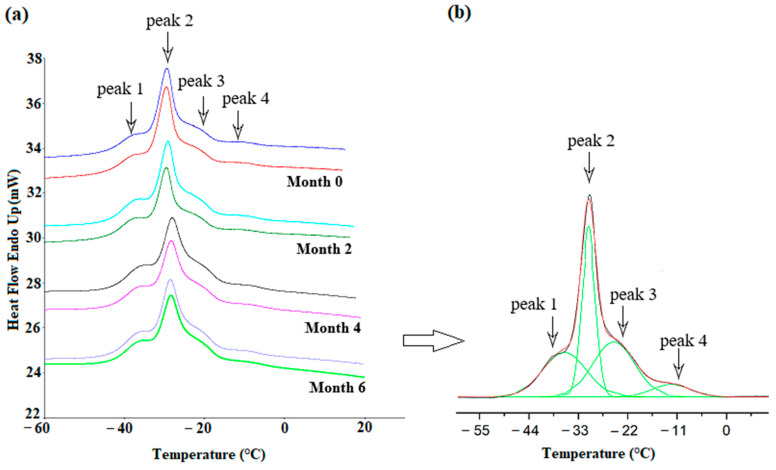
(**a**) Melting curves obtained from DSC analysis of cold-pressed flaxseed oil (FL_SZB) with heating rate of 5 °C/min during whole shelf life (0, 2, 4, and 6 months); (**b**) and deconvolution of melting curve of fresh flaxseed oil.

A DSC analysis of the melting profiles was carried out for all the flaxseed oil varieties throughout the shelf life (0, 2, 4, and 6 months) to determine the stability of the thermodynamic parameters. Figure 2a illustrates the endothermic curves generated from the phase transition of melting a flaxseed oil sample of the Szafir variety. The melting process for the samples was followed by prior crystallization of the samples at −65 °C with cooling at a scanning rate of 2 °C/min, after which the sample was heated with a scanning rate of 5 °C/min. Due to the complexity of the melting curves, which appear to be multicomponent, as the plethora of lipid components melt sequentially according to their increasing melting point, there was a need to use a peak deconvolution procedure, as shown in Figure 2b. For all the varieties, four peaks were identified during the melting process occurring from −65 °C to 30 °C. From the DSC curves presented in Figure 2, it is clear that the melting temperature initiated at around −40 °C, and the phase transition finished after the formation of the four encompassing peaks until the temperature reached around 0 °C. Among these four peaks, the second was considered to be the major peak, which occurred at around −30 °C. There are three more shoulder peaks along with the second peak, where the first peak can be detected at around −36 °C, and the comparatively smaller third and fourth peaks appeared at temperatures of approx. −25 °C and −12 °C, respectively. The melting behavior of the flaxseed oil is influenced by the composition of fatty acids that are bound in acylglycerols, mainly DAG and TAG. As was shown in a lipidomic study, 39 DAGs and 110 TAGs in the flaxseed oil were identified [32], which differ in their melting points. The position of the unsaturated bonds and the length of the fatty acid chains regulates the energy involved in the phase transition of the polymorph [33]. The appearance of multiple peaks can be the consequence of two simultaneously occurring phenomena, i.e., the complex distribution of TAGs and the polymorphic rearrangement of crystals, which can especially take place during the slow melting of lipid components [34]. In this study, a fast-scanning rate (5 °C/min) was used, which limits the polymorphic transitions. Furthermore, the distribution of the TAG’s as polymorphs cannot be surmised using the DSC curves, and research rather suggests that the X-ray diffraction method might be an effective tool to understand the in-depth cognition of each crystal packed in a TAG molecule and their discrete melting behavior [35,36]. However, the DSC curves can provide us with the paradigm of heat transition from one physical state to another [37]. 

In order to assess the parameters describing the melting curves, a deconvolution procedure was carried out to separate the four peaks. Over the decades, a computational method using a deconvolution algorithm has been employed by authors to analyze the complex DSC profiles [38,39]. Several parameters were taken under consideration to access the melting phase transition occurring in the flaxseed oil samples, i.e., peak temperature, peak height, peak area, and the percentage of the area. As a result, the various parameters of each peak were obtained, i.e., peak temperatures (T1, T2, T3, and T4), which are shown in Table 2; the peak heights (h1, h2, h3, and h4), which are presented in Table 3; the peak areas (A1, A2, A3, and A4); and the calculated percentages of the peak areas (P A1, P A2, P A3, and P A4), which are shown in Table 4.

Table 2 presents the peak temperature data of the four peaks identified for the five fresh and stored (2, 4, and 6 months) flaxseed oils samples. Four endothermic peaks appeared around the same temperature range for all varieties, which confirms the similarity of the thermal profiles for the flaxseed oils. The statistical exploration was based on a comparison of the variances between the varieties and between the different storage times. Generally, the comparison of varieties for the fresh oils showed that the temperatures of peaks T2, T3, and T4 were significantly higher only for sample FL_SZB than for the rest of the samples (*p* ≤ 0.05), although these differences were not greater than 2 °C. Temperature peak differences among the varieties are associated with the composition of PUFA, MUFA, and SFA in the flaxseed oils. As shown in Table 2, samples FL_SZB and FL_NN were characterized by the lowest content of PUFA, which justifies the highest peak temperatures for those varieties with a lower count of unsaturated C=C bonds (i.e., MUFA and PUFA). Other authors also stated that for canola oil, those peaks, which represent a lower temperature transition, are associated with the melting of unsaturated fatty acids [30]. Additionally, four peak temperatures throughout the storage time (0, 2, 4, and 6 months) were compared, as presented in Table 2. These data show that the main peak (T2) always gradually shifted towards a higher temperature until six months of storage (significantly, *p* ≤ 0.05), except for the FL_NN sample, for which the parameter T2 increased with time, but the changes were not significant (*p* > 0.05). The peak temperature T4 was significantly higher for all oil samples after six months (*p* ≤ 0.05). In comparison, T1 and T3 seemed to be more stable throughout the storage time analysis, since no significant changes took place for the measurements taken in different months (*p* > 0.05).

Table 3 presents the results of the measurement of the peak height of the four peaks calculated from the DSC curves. The parameter of the peak height expresses in the DSC analysis the intensity of the phase transition phenomena, which is dependent on the scanning rate used; the higher the scanning rate, the higher the peak height [2]. Thus, the parameters of the peak height that were obtained with the same scanning rate can only be compared, since the peak height is proportional to the rate of the heat transfer, which was also confirmed by other authors [40]. Generally, the parameters of the peak height (h1, h2, h3, and h4) for the fresh flaxseed oils did not differ significantly between the oil varieties, except for sample FL_BU, for which h1 was significantly different from the rest of the oil varieties (*p* ≤ 0.05). Among all the flaxseed oils samples, the peak height of the main peak (h2) and the last peak (h4) were significantly (*p* ≤ 0.05) lowered after six months of storage. On the other hand, two other minor peaks, h1 and h3, were quite stable during the storage period.

Table 4 presents the results of the calculations of the peak area of the four peaks (A1, A2, A3, and A4) determined by the deconvolution analysis. Comparing the parameters of the peak area between the oil samples, it can be seen that the area of the second peak (A2), determined between 2.2 and 2.7 for the fresh oils, makes the greatest contribution to the melting phenomena of the complex TAG structures. For the fresh flaxseed oils, A2 did not differ significantly between the oil varieties (*p* > 0.05). 

Analyzing the influence of the storage time, it can be seen that the area of the first and third peaks (A1, A3) did not change significantly within the six months of storage, in contrast to the values for the second (A2) and fourth (A4) peaks, for which a significant lowering of values was observed within the storage time (*p* ≤ 0.05). It is noticeable that the changes in the area of the peaks (A2, A4) observed during storage are in line with the changes in the peak height values (h2, h4). As shown in Table 4, the percentage of each peak area was also calculated based on the accumulated values of the total area of the endothermic peaks of the melting curves. Comparing the values of the percentage of the area within the storage time, it can be seen that for the first, second, and third peaks, the parameters P A1 and P A3 increased significantly (*p* ≤ 0.05), while PA2 decreased after six months. This is because the values of the peak areas (A1, A2, A3, and A4) are expressed as absolute values and the percentages of the area (P A1, P A2, P A3, P A4) are relative values, depending on the changes in the area of other peaks. The difference between the area and the percentage of the area is visible in the example of the first and third peaks (A1 and A3), which did not change significantly within the six months of storage, whereas the percentage of the peak area (P A1, P A3) increased, due to the decrease in the area of the second peak (A2). This observation implies that the changes in the percentage of the area (P A) should be considered only when all the peaks are analyzed together.

## 4. Discussion

### 4.1. Determination of DSC Parameters as Markers of Authenticity and Deterioration of Flaxseed Oil

For the purposes of this study, control charts were used to select the DSC parameters which remained stable throughout the shelf-life period, which can be used for fingerprinting as authenticity markers. On the other hand, all DSC parameters changing within the time of storage can be recognized as indicators of deterioration. Control charts are a common statistical tool for monitoring the conformity of products or processes with a reference value [41,42]. Referring to the study conducted on the stability of the DSC profile, monitoring with a control chart means that if the storage time does not affect the changes of the selected parameters, its level will not exceed the control limits (±3σ), and it will then be considered as a stable parameter during storage. Therefore, it appears reasonable to suggest the possibility of determining the limits that these parameters can reach regardless of the storage time of the oils. They will be characteristic for the different types of oils. In this study, X-bar and R (arithmetic mean and range) control charts were used to test the stability of the selected melting profile parameters. The X-bar chart and the R chart are actually two different graphs that must be considered in tandem to understand the behavior of the parameter being measured. The X-bar chart shows the average level of the parameter being monitored, while the R chart shows the range, i.e., the difference between the smallest and largest value in each sample, at each time point of storage, thus explaining the variability of the variation. In fact, to the best of the authors’ knowledge, there is no published research where a control chart was used to monitor the changes in the parameters within the storage time of cold-pressed oils. Using control charts, 16 variables were determined from the melting curves (T, h, A, and P A for the four peaks), and 18 variables were determined as ratios calculated for parameters h, A, and P A and were tested by using X-bar charts and the R charts.

#### 4.1.1. Determination of Stable DSC Parameters as Markers of Oils’ Authenticity during Storage

**Figure 3:**
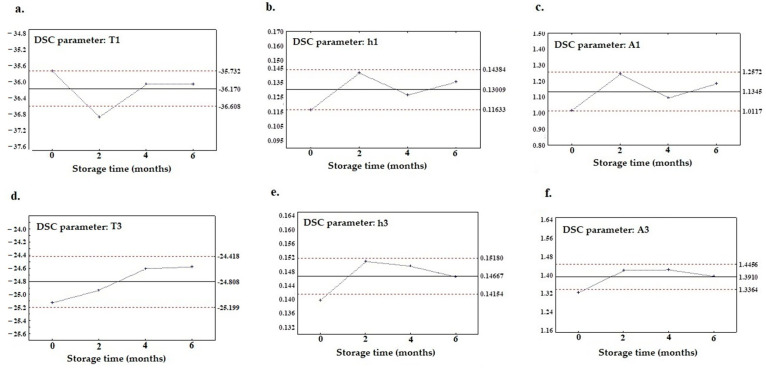
X-bar control charts of DSC parameters recognized as stable within six months storage; for the first peak: (**a**) T1, (**b**) h1, and (**c**) A1, and for the third peak: (**d**) T3, (**e**) h3, and (**f**) A3.

As it was shown in Table 2, Table 3 and Table 4, various parameters of the melting curve for flaxseed oils, i.e., peak temperature (T1, T2, T3, and T4), peak height (h1, h2, h3, and h4), peak area (A1, A2, A3, and A4), and the percentage of the peak area (P A1, P A2, P A3, and P A4) were analyzed within six months of storage. It was shown that for the first and third peaks, all DSC parameters (T, h, A, P A) did not change significantly within the storage time (*p* > 0.05). From a total amount of 16 variables (4 DSC parameters × 4 peaks) analyzed, the parameters that were remaining and anchored within the control limit in the X-bar chart were selected and denoted as ‘stable parameters’. Figure 3 shows that the control charts for these parameters were found to be stable during storage, which are pertinent for all oil samples. No point exceeded the control lines on either the X-bar charts or on the R charts between “0 month” and “6 months” of storage. This means that the average level of the parameter, as well as its variability, was stable throughout the storage period. The control limits on the X-bar charts can therefore be considered as a range that is characteristic and not changeable during storage for a particular melting profile parameter of the flaxseed oil. In Figure 3a–c, stable parameters associated with the first peak are presented (i.e., T1, h1, and A1), while Figure 3d–f illustrates the parameters for the third peak (i.e., T3, h3, and A3). Based on the data acquired from the melting curves, these multivariate X-bar charts show that the first and third peak areas, heights, and temperatures did not cross the limits between the upper control limit line (ULC) and the lower control limit line (LLC), except for the T1, h3, and A3 parameters, where one control point appeared outside the range. 

**Figure 4:**
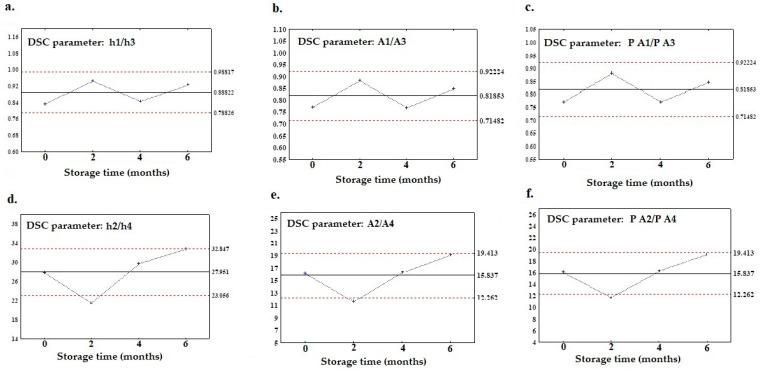
X-bar control charts of DSC parameters recognized as stable within six months of storage; for the ratio calculated for the first and third peaks: (**a**) h1/h3, (**b**) A1/A3, and (**c**) P A1/P A3, and for the second and fourth peaks: (**d**) h2/h4, (**e**) A2/A4P, and (**f**) P A2/P A4.

However, after careful observation of those points, it can be seen that the differences between the ULC and LLC are extraordinarily minimal compared to the range of undisputed parameters like A1, h1, and T3. Meanwhile, Figure 4a–f also shows a graph with the X-bar control charts prepared on the basis of the calculated ratio of DSC parameters. It can be observed that in addition to the ratio of parameters calculated for the first and third peaks, i.e., h1/h3, A1/A3, and P A1/P A3 (Figure 4a–c), there are other parameters fitting to the control limits, such as h2/h4, A2/A4, and P A2/PA4, which are calculated for the second and fourth peak (Figure 4d–f). The pivotal role of the first and third peaks regarding the authenticity analysis can be seen, and the ratio of the parameters for these two peaks are also persistently framed within the control limits. Furthermore, Figure 4 shows that the ratios calculated for the second and fourth peaks also abide by the rules to be considered as the stable parameters (h2/h4, A2/A4, and P A2/P A4), despite the fact that these individually considered parameters were not stable. It is worth noting that the parameters accountable for the percentage of the area (P A) do not have coherent properties when analyzed separately, since they depend on the other peaks, but the ratio calculated from parameters P A appeared to be a better option in this respect.

#### 4.1.2. Detection of Flaxseed Oil Deterioration by Unstable DSC Parameters during Storage

**Figure 5:**
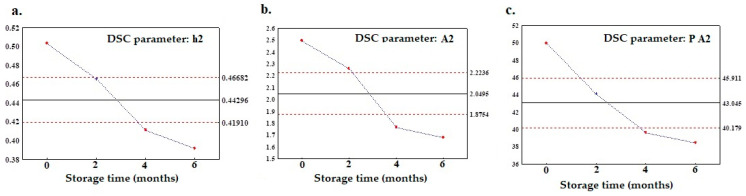
X-bar control charts of unstable parameters calculated from DSC melting curves for the second peak: (**a**) h2, (**b**) A2, and (**c**) P A2.

The second purpose of this study was to recognize the DSC melting profile parameters that changed within the storage time. However, only the parameters with increasing or decreasing trends indicating changes caused by storage were considered. In order to establish these, the control charts were analyzed in terms of the parameters for which the two control lines for the fresh and stored oils were exceeded via crossing the ULC and LLC from both ends. Figure 5a–c illustrates the control charts with the parameters of the main peak height and area (h2, A2, and PA2) for which the values decrease throughout the shelf life of the oil. From Table 3 and Table 4, it is clearly seen that for all the flaxseed oil varieties, the values h2, A2, and P A2 are significantly lower after six months of storage, which coincides with the control charts presented in Figure 5a–c.

**Figure 6:**
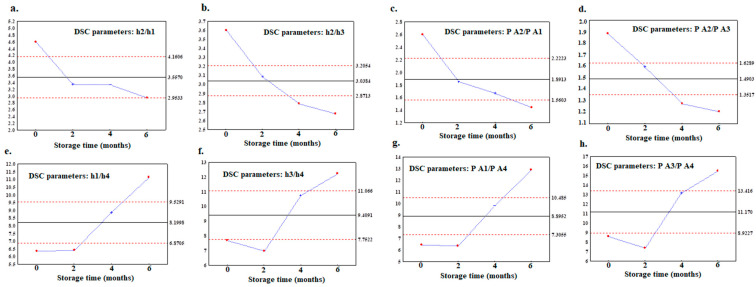
X-bar control charts of ratios calculated from DSC melting curves with downward trends: (**a**) h2/h1, (**b**) h2/h3, (**c**) A2/A1, (**d**) A2/A3, and with upward trends: (**e**) h1/h4, (**f**) h3/h4, (**g**) A1/A4, and (**h**) A3/A4.

The trend within the storage time of the ratio calculated for the parameters of the peak height (h2/h1, h2/h3, h1/h4, and h3/h4), as well as the ratio of the peak area (A2/A1, A2/A3, A1/A4, and A3/A4) was also analyzed using the control charts (Figure 6a–h). can be seen, a downward trend was observed for ratios h2/h1 and h2/h3 (Figure 6a,b), as well as for A2/A1 and A2/A3 (Figure 6c,d). In turn for the parameters of h1/h4, h3/h4, A1/A4, and A3/A4, an increasing trend was shown (Figure 6e–h). Analogously, as for the ratios of the peak area (A2/A1, A2/A3, A1/A4, and A3/A4), the same trend was observed for the ratio of the percentage of the area (PA2/PA1, PA2/PA3, PA1/PA4, and PA3/PA4), which means that the values increased or decreased by the same pattern (Figure S1; Supplementary Data). 

**Figure 7:**
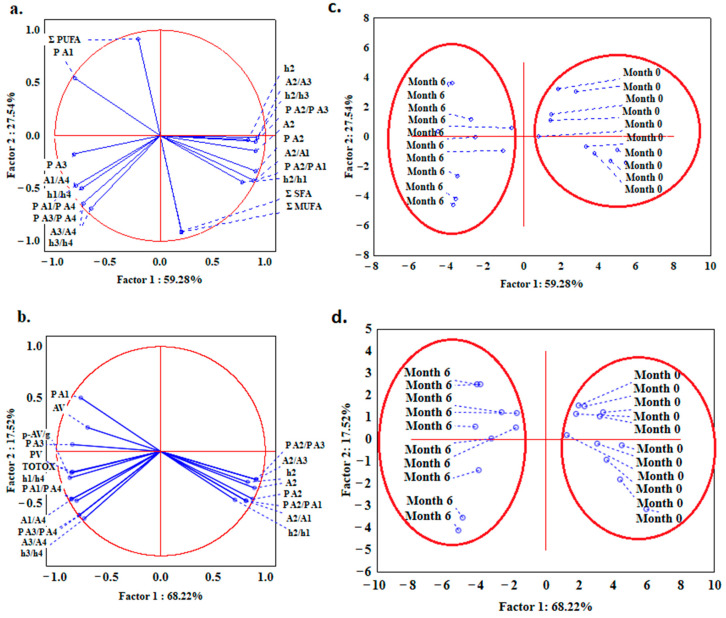
PCA analysis with projection of the variables: (**a**) DSC unstable parameters and fatty acids content; (**b**) DSC unstable parameters and chemical indicators (PV, p-AV, AV, and TOTOX); PCA analysis with projection of the cases showing distribution and separation of flaxseed oil samples based on their stability control by means of (**c**) DSC parameters and fatty acids content; and (**d**) DSC parameters and chemical indicators.

In addition, a PCA analysis was applied with all the unstable parameters obtained from the DSC and fatty acid content (∑SFA, ∑MUFA, ∑PUFA) (Figure 7a,c); and also, for the DSC unstable parameters and chemical indicators (PV, p-AV, AV, TOTOX) (Figure 7b,d) for the fresh oil samples and the ones that were stored for 6 months. It was shown that for both approaches, two distinctive clusters were separated, with a differentiation between the two groups of oils, i.e., fresh and stored (Figure 7c,d). In the graph of the variable projection (Figure 7a,b), it can be seen that the parameters connected with the second peak (h2, A2, and P A2) were the highest for fresh oil and the lowest after 6 months, in contrast to the parameters of the ratios calculated for the first and fourth peaks, as well as for the third and fourth peaks. Chemical indicators of the oxidative stability of the oil (PV, p-AV, AV, and TOTOX) were found to have strong negative correlations with the DSC unstable parameters connected to the second peak of the melting profile (for instance h2, A2, and P A2). Significant correlations (*p* ≤ 0.05) were observed between p-AV, PV, AV, TOTOX, and DSC parameters that were determined for the second peak, as the Pearson correlation coefficients were, e.g., for h2: −0,63, −0.64, −0.57, and −0.64, and for A2: −0.51, −0.51, −0.64, and −0.55, respectively.

## 5. Conclusions

In the age of the global food market, rapid and ecological methods which will facilitate a fast authentication assessment are crucial. At the same level, controlling the quality of flaxseed oil is important, since it is highly susceptible to the oxidation process occurring during prolonged storage. Chemical analysis of the oxidative stability (AV, PV, p-AV, and TOTOX) showed the changes occurring during the storage of flaxseed oil for six months, since all parameters measured increased for all the oil samples. This study proposed an approach using X-bar and R control charts to monitor the stability and changes of the DSC melting profiles of flaxseed oil stored for six months in conditions similar to the supermarket shelf. By implementing the procedure of deconvolution, four peaks were identified in the melting profile, for which the peak temperature, peak height, peak area, and the percentage of peak area were analyzed. Analysis by means of control charts enabled stable parameters of the melting profile of flaxseed oil to be determined, which were connected to the first and third peak. In total, twelve parameters calculated based on those peaks were selected, which can be considered as indicators for authentication. On the other hand, it was also possible to indicate from the melting profile those parameters that changed throughout the shelf life with an increasing or decreasing tendency. Those parameters were mainly connected with the major second peak, as well as with the ratios calculated for the first to fourth and third to fourth peak. Sixteen parameters calculated from the melting profile were found to be unstable parameters, which can be used as indicators for the deterioration of oil. In the case of the percentage of peak area (PA), values of this parameter should be considered all together, since they depend on the other peaks. Strong negative correlations of the DSC unstable parameters with chemical indicators of the oils’ oxidative stability were found. 

## Figures and Tables

**Table 1:** Acid values (AV), peroxide values (PV), p-anisidine values (p-AV), TOTOX values for cold- pressed flaxseed oils at fresh condition and after 6 months storage time.

Seeds Varieties	Time	Chemical Analysis			
		AV (mg KOH/g)	PV (meq O2/kg)	p-AV	TOTOX
FL_BU	0 month	0.80 ± 0.04 ^b^	3.95 ± 0.63 ^c^	0.87 ± 0.35 ^a^	8.78 ± 1.02 ^c^
	After 6 months	2.54 ± 0.02 ^g^	14.44 ± 0.08 ^h^	2.02 ± 0.02 ^c^	30.90 ± 0.15 ^h^
FL_DL	0 month	0.41 ± 0.01 ^a^	2.49 ± 0.15 ^b^	0.65 ± 0.01 ^a^	5.63 ± 0.31 ^b^
	After 6 months	1.59 ± 0.02 ^d^	10.64 ± 0.11 ^f^	1.70 ± 0.07 ^bc^	22.76 ± 0.21 ^f^
FL_SZA	0 month	1.27 ± 0.03 ^c^	1.41 ± 0.06 ^a^	0.73 ± 0.13 ^a^	3.54 ± 0.20 ^a^
	After 6 months	2.40 ± 0.02 ^f^	9.73 ± 0.13 ^e^	1.49 ± 0.01 ^b^	21.09 ± 0.28 ^e^
FL_SZB	0 month	0.81 ± 0.03 ^b^	6.90 ± 0.23 ^d^	0.94 ± 0.12 ^a^	14.74 ± 0.41 ^d^
	After 6 months	1.79 ± 0.02 ^e^	15.57 ± 0.16 ^i^	1.63 ± 0.02 ^bc^	33.61 ± 0.30 ^i^
FL_NN	0 month	1.60 ± 0.02 ^d^	1.21 ± 0.06 ^a^	0.76 ± 0.25 ^a^	3.17 ± 0.24 ^a^
	After 6 months	3.25 ± 0.10 ^h^	11.69 ± 0.22 ^g^	2.47 ± 0.03 ^d^	25.08 ± 0.50 ^g^

All values are mean ± standard deviation of three measurements (n = 3); (a–i)—means with the same letters within the column are not different (*p* > 0.05).

**Table 2:** Peak temperature changes (T, °C) of DSC melting curves of flaxseed oils during storage (0, 2, 4, and 6 months).

Peak Temperature (°C)	Time	FL_BU	FL_DL	FL_SZA	FL_SZB	FL_NN
T1	0	−36.85 ± 0.11 ^aA^	−36.37 ± 0.12 ^aAB^	−36.65 ± 0.22 ^aAB^	−36.36 ± 0.07 ^aA^	−36.55 ± 0.93 ^aA^
	2	−37.19 ± 0.37 ^abA^	−36.93 ± 0.27 ^abcA^	−37.57 ± 0.36 ^aA^	−36.2 ± 0.18 ^cA^	−36.44 ± 0.32 ^bcA^
	4	−36.72 ± 0.62 ^aA^	−36.58 ± 0.06 ^aAB^	−36.44 ± 0.18 ^aB^	−35.69 ± 0.46 ^abA^	−34.80 ± 0.17 ^bB^
	6	−36.55 ± 0.18 ^aA^	−36.12 ± 0.29 ^abB^	−36.38 ± 0.20 ^aB^	−35.59 ± 0.11 ^bA^	−35.59 ± 0.06 ^bAB^
T2	0	−31.67 ± 0.09 ^aA^	−30.10 ± 0.11 ^abAB^	−30.37 ± 0.25 ^abB^	−29.72 ± 0.05 ^bA^	−30.01 ± 0.97^a bA^
	2	−31.54 ± 0.2 ^aA^	−30.50 ± 0.29 ^bcA^	−31.06 ± 0.21 ^abA^	−29.64 ± 0.23 ^dA^	−30.00 ± 0.25 ^cdA^
	4	−30.88 ± 0.27 ^aB^	−29.31 ± 0.28 ^bcB^	−29.53 ± 0.12 ^bC^	−28.35 ± 0.20 ^dB^	−28.66 ± 0.03 ^cdA^
	6	−30.22 ± 0.11 ^aB^	−29.39 ± 0.24 ^bB^	−29.67 ± 0.08 ^abC^	−28.6 ± 0.13 ^cB^	−28.55 ± 0.08 ^cA^
T3	0	−25.12 ± 0.00 ^aA^	−24.94 ± 0.18 ^abA^	−24.96 ± 0.2 ^abA^	−24.19 ± 0.12 ^bA^	−24.58 ± 0.54 ^abA^
	2	−25.01 ± 0.00 ^aA^	−25.05 ± 0.09 ^aA^	−25.14 ± 0.00 ^aA^	−24.85 ± 0.08 ^abA^	−24.55 ± 0.22 ^bA^
	4	−24.98 ± 0.16 ^aA^	−25.10 ± 0.00 ^aA^	−25.11 ± 0.00 ^aA^	−24.63 ± 0.66 ^aA^	−23.31 ± 0.16 ^bB^
	6	−25.12 ± 0.00 ^aA^	−24.55 ± 0.77 ^aA^	−25.13 ± 0.00 ^aA^	−24.47 ± 0.04 ^aA^	−22.99 ± 0.07 ^bB^
T4	0	−13.63 ± 0.24 ^aA^	−12.50 ± 0.49 ^abA^	−12.64 ± 0.34 ^abA^	−12.20 ± 0.06 ^bA^	−13.87± 0.18 ^aA^
	2	−13.57 ± 0.16 ^aA^	−12.84 ± 0.18 ^abA^	−13.27 ± 0.20 ^aA^	−11.76 ± 0.42 ^cA^	−12.21 ± 0.61 ^bcB^
	4	−14.02 ± 0.00 ^aA^	−10.62 ± 0.23 ^bcB^	−11.13 ± 0.24 ^bB^	−10.40 ± 0.29 ^bcB^	−9.94 ± 0.15 ^cC^
	6	−12.05 ± 0.24 ^bB^	−11.24 ± 0.16 ^aB^	−11.13 ± 0.04 ^abB^	−10.54 ± 0.28 ^cB^	−10.49 ± 0.08 ^cC^

FL_BU (Bukoz cultivar), FL_DL (Dolguniec cultivar), FL_SZA, FL SZB (Szafir cultivar), FL_NN (Unknown Flaxseed cultivar). T1—peak temperature for first peak, counting from lower to higher temperature as T2, T3, and T4. All values are mean ± standard deviation of the two replicates. (a–d) means with the same superscript within the same row are not different (*p* > 0.05); (A–C) means with the same superscript within the same column are not different (*p* > 0.05).

**Table 3:** Peak height changes (h, W/g) of DSC melting curves of flaxseed oils during storage (0, 2, 4, and 6 months).

Peak Height (W/g)	Time	FL_BU	FL_DL	FL_SZA	FL_SZB	FL_NN
h1	0	0.15 ± 0.008 ^bB^	0.13 ± 0.008 ^abA^	0.14 ± 0.005 ^abA^	0.12 ± 0.008 ^aA^	0.08 ± 0.014 ^aA^
	2	0.17 ± 0.007 ^cB^	0.15 ± 0.006 ^bA^	0.15 ± 0.008 ^bcA^	0.13 ± 0.004 ^aA^	0.12 ± 0.003 ^aA^
	4	0.12 ± 0.004 ^abcA^	0.14 ± 0.008 ^bcA^	0.15 ± 0.002 ^cA^	0.11 ± 0.006 ^abA^	0.11 ± 0.013 ^aA^
	6	0.15 ± 0.006 ^aB^	0.13 ± 0.024 ^bA^	0.15 ± 0.011 ^abA^	0.12 ± 0.000 ^cA^	0.12 ± 0.001 ^cA^
h2	0	0.47 ± 0.033 ^aB^	0.52 ± 0.099 ^aA^	0.46 ± 0.003 ^aB^	0.52 ± 0.002 ^aC^	0.51 ± 0.062 ^aAB^
	2	0.43 ± 0.016 ^aB^	0.46 ± 0.008 ^abA^	0.46 ± 0.012 ^abB^	0.48 ± 0.013 ^bB^	0.48 ± 0.031 ^bB^
	4	0.45 ± 0.005 ^cB^	0.37 ± 0.005 ^abA^	0.37 ± 0.000 ^aA^	0.40 ± 0.005 ^bA^	0.46 ± 0.016 ^cAB^
	6	0.36 ± 0.007 ^aA^	0.42 ± 0.040 ^aA^	0.39 ± 0.013 ^aA^	0.41 ± 0.010 ^aA^	0.38 ± 0.000 ^aA^
h3	0	0.14 ± 0.006 ^aA^	0.14 ± 0.006^a A^	0.14 ± 0.000 ^aA^	0.15 ± 0.005 ^aA^	0.13 ± 0.000 ^aA^
	2	0.14 ± 0.002 ^aA^	0.15 ± 0.006 ^bAB^	0.15 ± 0.001 ^bB^	0.16 ± 0.006 ^bA^	0.16 ± 0.002 ^bB^
	4	0.14 ± 0.003 ^aA^	0.17 ± 0.003 ^bB^	0.16 ± 0.008 ^abB^	0.15 ± 0.010 ^abA^	0.14 ± 0.008 ^aA^
	6	0.14 ± 0.001 ^aA^	0.14 ± 0.002 ^aA^	0.15 ± 0.001 ^aB^	0.15 ± 0.008 ^aA^	0.14 ± 0.001 ^aA^
h4	0	0.02 ± 0.001 ^cAB^	0.01 ± 0.001 ^aA^	0.02 ± 0.001 ^bAB^	0.02 ± 0.001 ^bcB^	0.02 ± 0.002 ^bcB^
	2	0.03 ± 0.002 ^cB^	0.02 ± 0.001 ^abA^	0.02 ± 0.001 ^bcB^	0.02 ± 0.001 ^aB^	0.02 ± 0.003 ^abB^
	4	0.02 ± 0.002 ^bA^	0.02 ± 0.001 ^abA^	0.02 ± 0.000 ^abA^	0.01 ± 0.002 ^bA^	0.01 ± 0.002 ^bA^
	6	0.02 ± 0.000 ^bA^	0.01 ± 0.004 ^abA^	0.01 ± 0.003 ^abA^	0.01 ± 0.003 ^abA^	0.01 ± 0.000 ^aA^

FL_BU (Bukoz cultivar), FL_DL (Dolguniec cultivar), FL_SZA, FL_SZB (Szafir cultivar), FL_NN (Unknown cultivar). h1—peak height for first peak, counting from first to fourth peak as h2, h3, and h4. All values are mean ± standard deviation of the two replicates. (a–c) means with the same superscript within the same row are not different (*p* > 0.05); (A–C) means with the same superscript within the same column are not different (*p* > 0.05).

**Table 4:** Peak area (A) and percentage of peak area (P A) changes of DSC melting curves for flaxseed oils during storage (0, 2, 4, and 6 months).

Peak Area	Time	FL_BU	FL_DL	FL_SZA	FL_SZB	FL_NN
A1	0	1.39 ± 0.07 ^bB^	1.18 ± 0.07 ^abA^	1.19 ± 0.04 ^abA^	1.03 ± 0.07 ^aA^	0.86 ± 0.12 ^aA^
	2	1.45 ± 0.06 ^cB^	1.27 ± 0.05 ^bA^	1.33 ± 0.07 ^bcA^	1.12 ± 0.04 ^aA^	1.04 ± 0.02 ^aA^
	4	1.09 ± 0.03 ^abA^	1.14 ± 0.1 ^abA^	1.30 ± 0.02 ^bA^	0.10 ± 0.07 ^aA^	0.93 ± 0.11 ^aA^
	6	1.36 ± 0.05 ^aB^	1.12 ± 0.21 ^aA^	1.30 ± 0.04 ^aA^	1.07 ± 0.00 ^aA^	1.08 ± 0.01 ^aA^
A2	0	2.50 ± 0.20 ^aB^	2.68 ± 0.73 ^aB^	2.22 ± 0.01 ^aB^	2.70 ± 0.05 ^aC^	2.42 ± 0.04 ^aA^
	2	2.20 ± 0.08 ^aB^	2.19 ± 0.04 ^aAB^	2.28 ± 0.03 ^aB^	2.21 ± 0.14 ^aB^	2.42 ± 0.26 ^aA^
	4	2.29 ± 0.04 ^bB^	1.23 ± 0.11 ^aA^	1.40 ± 0.09 ^aA^	1.61 ± 0.05 ^aA^	2.31 ± 0.20 ^bA^
	6	1.41 ± 0.06 ^aA^	1.74 ± 0.16 ^abAB^	1.62 ± 0.18 ^abA^	1.72 ± 0.05 ^abA^	1.89 ± 0.02 ^bA^
A3	0	1.20 ± 0.00 ^aA^	1.35 ± 0.06 ^abA^	1.32 ± 0.00 ^abA^	1.44 ± 0.05 ^bA^	1.25 ± 0.00 ^aA^
	2	1.29 ± 0.03 ^aB^	1.44 ± 0.06 ^bAB^	1.41 ± 0.06 ^abA^	1.50 ± 0.06 ^bA^	1.48 ± 0.02 ^bB^
	4	1.29 ± 0.02 ^aB^	1.58 ± 0.02 ^bB^	1.49 ± 0.08 ^abA^	1.44 ± 0.09 ^abA^	1.32 ± 0.08 ^bA^
	6	1.38 ± 0.01 ^aC^	1.38 ± 0.02 ^aA^	1.47 ± 0.01 ^aA^	1.41 ± 0.08 ^aA^	1.34 ± 0.01 ^aA^
A4	0	0.21 ± 0.01 ^bB^	0.14 ± 0.02 ^aAB^	0.18 ± 0.02 ^abB^	0.18 ± 0.02 ^abB^	0.22 ± 0.02 ^bB^
	2	0.22 ± 0.01 ^bB^	0.19 ± 0.01 ^abB^	0.23 ± 0.01 ^bC^	0.15 ± 0.02 ^aB^	0.20 ± 0.04 ^abB^
	4	0.18 ± 0.02 ^bAB^	0.12 ± 0.01 ^aA^	0.13 ± 0.00 ^abA^	0.08 ± 0.02 ^aA^	0.08 ± 0.02 ^aA^
	6	0.14 ± 0.00 ^bA^	0.11 ± 0.02 ^abA^	0.11 ± 0.02 ^abA^	0.09 ± 0.02 ^abA^	0.06 ± 0.00 ^aA^
% peak area						
P A1	0	26.21 ± 0.07 ^bB^	22.18 ± 2.33 ^abA^	24.25 ± 0.57 ^abA^	19.23 ± 0.99 ^aA^	18.16 ± 2.17 ^aA^
	2	28.16 ± 1.30 ^cB^	25.02 ± 0.20 ^bA^	25.33 ± 1.09 ^bcA^	22.54 ± 0.74 ^abB^	20.33 ± 1.62 ^aA^
	4	22.49 ± 0.13 ^aA^	28.01 ± 1.06 ^bcA^	30.20 ± 0.25 ^cB^	24.11 ± 1.49 ^abB^	20.13 ± 2.37 ^aA^
	6	31.65 ± 0.36 ^bC^	25.58 ± 2.89 ^aA^	28.97 ± 0.47 ^abB^	24.93 ± 0.04 ^aB^	24.67 ± 0.15 ^aA^
P A2	0	48.21 ± 1.32 ^aC^	50.03 ± 5.48 ^aC^	45.91 ± 0.42 ^aB^	52.65 ± 1.66 ^aC^	51.71 ± 1.92 ^aA^
	2	42.67 ± 1.12 ^aB^	43.03 ± 0.59 ^aBC^	43.40 ± 0.70 ^aB^	44.35 ± 0.95 ^abB^	47.08 ± 2.32 ^bA^
	4	47.15 ± 0.22 ^bcC^	30.07 ± 1.38 ^aA^	32.45 ± 1.88 ^aA^	38.94 ± 0.73 ^abA^	49.70 ± 4.3 ^cA^
	6	32.99 ± 0.62 ^aA^	40.01 ± 0.65 ^bcB^	35.88 ± 2.1 ^abA^	40.12 ± 1.26 ^bcA^	43.37 ± 0.45 ^cA^
P A3	0	22.68 ± 1.15 ^aA^	25.49 ± 3.05 ^aA^	26.85 ± 0.41 ^aA^	26.85 ± 0.45 ^aA^	26.36 ± 0.49 ^aA^
	2	24.87 ± 0.47 ^aAB^	28.21 ± 0.24 ^bcA^	26.87 ± 1.32 ^abA^	30.03 ± 0.41 ^cAB^	28.80 ± 1.28 ^abAB^
	4	26.63 ± 0.26 ^aB^	38.92 ± 2.47 ^cB^	34.43 ± 2.14 ^abcB^	34.95 ± 2.58 ^bcB^	28.35 ± 1.59 ^abAB^
	6	32.18 ± 0.98 ^aC^	31.80 ± 2.79 ^aAB^	32.77 ± 1.96 ^aB^	32.86 ± 1.88 ^aB^	30.68 ± 0.24 ^aB^
P A4	0	3.94 ± 0.10 ^bcAB^	2.61 ± 0.11 ^aA^	3.67 ± 0.26 ^bcB^	3.45 ± 0.22 ^abB^	4.55 ± 0.25 ^cB^
	2	4.30 ± 0.24 ^bB^	3.74 ± 0.16 ^abA^	4.40 ± 0.14 ^bC^	3.07 ± 0.20 ^aAB^	3.78 ± 0.61 ^abB^
	4	3.73 ± 0.36 ^cAB^	2.99 ± 0.03 ^bcA^	2.92 ± 0.01 ^abcAB^	1.99 ± 0.39 ^abA^	1.81 ± 0.34 ^aA^
	6	3.17 ± 0.01 ^bA^	2.61 ± 0.76 ^abA^	2.37 ± 0.33 ^abA^	2.09 ± 0.58 ^abA^	1.27 ± 0.06 ^aA^

FL_BU (Bukoz cultivar), FL_DL (Dolguniec cultivar), FL_SZA, FL SZB (Szafir cultivar), FL_NN (Unknown cultivar). A1—peak area for first peak, counting from first to fourth peak as A2, A3, and A4. and P A1—percentage of peak area for first peak, counting from first peak to fourth peak as P A2, P A3, and P A4. All values are mean ± standard deviation of the two replicates. (a–c) means with the same superscript within the same row are not different (*p* > 0.05); (A–C) means with the same superscript within the same column are not different (*p* > 0.05).

## Data Availability

Data is contained within the article and supplementary material.

## Supplementary Materials

The following supporting information can be downloaded at: https://www.mdpi.com/article/10.3390/foods12152954/s1, Figure S1: Control charts of unstable parameters (calculated from the ratio of peak’s percentage of area) from DSC melting curves. (a) Ratio of parameters P A2/P A1, (b) ratio of parameters P A2/P A3, (c) ratio of parameters P A1/P A4, and (d) ratio of parameters P A3/P A4.

## References

[1] FAO Production/Yield Quantities of Oil of Linseed in World + (Total). https://www.fao.org/faostat/en/#data/QCL/visualize.

[2] Tomaszewska-Gras J., Islam M., Grzeca L., Kaczmarek A., Fornal E. (2021). Comprehensive Thermal Characteristics of Different Cultivars of Flaxseed Oil (*Linum usittatissimum* L.). Molecules.

[3] Obranović M., Škevin D., Kraljić K., Pospišil M., Neđeral S., Blekić M., Putnik P. (2015). Influence of Climate, Variety and Production Process on Tocopherols, Plastochromanol-8 and Pigments in Flaxseed Oil. Food Technol. Biotechnol..

[4] Herchi W., Bouali I., Bahashwan S., Rochut S., Boukhchina S., Kallel H., Pepe C. (2012). Changes in Phospholipid Composition, Protein Content and Chemical Properties of Fl Axseed Oil during Development. Plant Physiol. Biochem..

[5] Khattab R.Y., Zeitoun M.A. (2013). Quality Evaluation of Flaxseed Oil Obtained by Different Extraction Techniques. LWT Food Sci. Technol..

[6] Komartin R.S., Stroescu M., Chira N., Stan R., Stoica-Guzun A. (2021). Optimization of Oil Extraction from Lallemantia Iberica Seeds Using Ultrasound-Assisted Extraction. J. Food Meas. Charact..

[7] Teh S.S., Birch J. (2013). Physicochemical and Quality Characteristics of Cold-Pressed Hemp, Flax and Canola Seed Oils. J. Food Compos. Anal..

[8] El-Beltagi H.S., Salama Z., El-Hariri D.M. (2007). Evaluation of Fatty Acids Profile and the Content of Some Secondary Metabolites in Seeds of Different Flax Cultivars (*Linum usitatissimum* L.). Gen. Appl. Plant Physiol..

[9] Tavarini S., Castagna A., Conte G., Foschi L., Sanmartin C., Incrocci L., Ranieri A., Serra A., Angelini L.G. (2019). Evaluation of Chemical Composition of Two Linseed Varieties as Sources of Health-Beneficial Substances. Molecules.

[10] Gallardo M.A., Milisich H.J., Drago S.R., González R.J. (2014). Effect of Cultivars and Planting Date on Yield, Oil Content, and Fatty Acid Profile of Flax Varieties (*Linum usitatissimum* L.). Int. J. Agron..

[11] Choe E., Min D.B. (2006). Mechanisms and Factors for Edible Oil Oxidation. Compr. Rev. Food Sci. Food Saf..

[12] Choo W.S., Birch J., Dufour J.P. (2007). Physicochemical and Quality Characteristics of Cold-Pressed Flaxseed Oils. J. Food Compos. Anal..

[13] Hasiewicz-Derkacz K., Kulma A., Czuj T., Prescha A., Zuk M., Grajzer M., Łukaszewicz M., Szopa J. (2015). Natural Phenolics Greatly Increase Flax (*Linum usitatissimum*) Oil Stability. BMC Biotechnol..

[14] Islam M., Muzolf-Panek M., Fornal E., Tomaszewska-Gras J. (2022). DSC Isothermal and Non-Isothermal Assessment of Thermo-Oxidative Stability of Different Cultivars of Camelina Sativa L. Seed Oils. J. Therm. Anal. Calorim..

[15] Tańska M., Roszkowska B., Skrajda M., Grzegorz D. (2016). Commercial Cold Pressed Flaxseed Oils Quality and Oxidative Stability at the Beginning and the End of Their Shelf Life. J. Oleo Sci..

[16] Islam M., Bełkowska L., Konieczny P., Fornal E., Tomaszewska-Gras J. (2022). Differential Scanning Calorimetry for Authentication of Edible Fats and Oils—What Can We Learn from the Past to Face the Current Challenges?. J. Food Drug Anal..

[17] Islam M., Rajagukguk Y.V., Siger A., Tomaszewska-Gras J. (2023). Assessment of Hemp Seed Oil Quality Pressed from Fresh and Stored Seeds of Henola Cultivar Using Differential Scanning Calorimetry. Foods.

[18] Cichocki W., Kmiecik D., Baranowska H.M., Staroszczyk H., Sommer A., Kowalczewski P.Ł. (2023). Chemical Characteristics and Thermal Oxidative Stability of Novel Cold-Pressed Oil Blends: GC, LF NMR, and DSC Studies. Foods.

[19] Rajagukguk Y.V., Islam M., Tomaszewska-Gras J. (2023). Influence of Seeds’ Age and Clarification of Cold-Pressed Raspberry (*Rubus idaeus* L.) Oil on the DSC Oxidative Stability and Phase Transition Profiles. Foods.

[20] Zhang Z.S., Li D., Zhang L.X., Liu Y.L., Wang X.-D. (2014). Heating Effect on the DSC Melting Curve of Flaxseed Oil. J. Therm. Anal. Calorim..

[21] AOCS (1997). AOCS Official Method Preparations of Methyl Esters of Fatty Acids. https://www.coursehero.com/file/53583244/AOCS-Official-Method-Ce-2-66-Preparation-of-Methyl-Esters-of-Fatty-Acidspdf/.

[22] AOCS (2009). AOCS Official Method Acid Value. Cd 3d-63. https://myaccount.aocs.org/PersonifyEbusiness/Store/Product-Details/productId/111545.

[23] (2017). Animal and Vegetable Fats and Oils—Determination—Iodometric (Visual) Endpoint.

[24] (2016). Animal and Vegetable Fats and Oils: Determination of Anisidine Value.

[25] Tomaszewska-Gras J. (2016). Rapid Quantitative Determination of Butter Adulteration with Palm Oil Using the DSC Technique. Food Control.

[26] Anastasiu A.E., Chira N.A., Banu I., Ionescu N., Stan R., Rosca S.I. (2016). Oil Productivity of Seven Romanian Linseed Varieties as Affected by Weather Conditions. Ind. Crop. Prod..

[27] Cantele C., Bertolino M., Bakro F., Giordano M., Jędryczka M., Cardenia V. (2020). Antioxidant Effects of Hemp (*Cannabis Sativa* L.) Inflorescence Extract in Stripped Linseed Oil. Antioxidants.

[28] Malcolmson L.J., Przybylski R., Daun J.K. (2000). Storage Stability of Milled Flaxseed. JAOCS J. Am. Oil Chem. Soc..

[29] Prescha A., Grajzer M., Dedyk M., Grajeta H. (2014). The Antioxidant Activity and Oxidative Stability of Cold-Pressed Oils. JAOCS J. Am. Oil Chem. Soc..

[30] Codex Alimentarius (1999). Standard for Named Vegetable Oils Codex Stan 210-1999. Codex Alimentarius.

[31] Gordon M.H. (2001). The Development of Oxidative Rancidity in Foods.

[32] Kozub A., Nikolaichuk H., Przykaza K., Tomaszewska-Gras J., Fornal E. (2023). Lipidomic Characteristics of Three Edible Cold-Pressed Oils by LC/Q-TOF for Simple Quality and Authenticity Assurance. Food Chem..

[33] Mcelhaney R.N. (1982). The Use of Differential Scanning Calorimetry and Differential Thermal Analysis in Studies of Model and Biological Membranes. Chem. Phys. Lipids.

[34] Tan C.P., Che Man Y.B. (2002). Comparative Differential Scanning Calorimetric Analysis of Vegetable Oils: I. Effects of Heating Rate Variation. Phytochem. Anal..

[35] Minato A., Ueno S., Yano J., Smith K., Seto H., Amemiya Y., Sato K. (1997). Thermal and Structural Properties of Sn-1,3-Dipalmitoyl-2-Oleoylglycerol and Sn-1,3-Dioleoyl-2-Palmitoylglycerol Binary Mixtures Examined with Synchrotron Radiation X-Ray Diffraction. JAOCS J. Am. Oil Chem. Soc..

[36] Pratama Y., Burholt S., Baker D.L., Sadeghpour A., Simone E., Rappolt M. (2022). Polymorphism of a Highly Asymmetrical Triacylglycerol in Milk Fat: 1-Butyryl 2-Stearoyl 3-Palmitoyl-Glycerol. Cryst. Growth Des..

[37] Tan C.P., Che Man Y.B. (2000). Differential Scanning Calorimetric Analysis of Edible Oils: Comparison of Thermal Properties and Chemical Composition. JAOCS J. Am. Oil Chem. Soc..

[38] Konieczny P., Tomaszewska-Gras J., Andrzejewski W., Mikołajczak B., Urbańska M., Mazurkiewicz J., Stangierski J. (2016). DSC and Electrophoretic Studies on Protein Denaturation of Anodonta Woodiana (Lea, 1834). J. Therm. Anal. Calorim..

[39] Vega S., Garcia-Gonzalez M.A., Lanas A., Velazquez-Campoy A., Abian O. (2015). Deconvolution Analysis for Classifying Gastric Adenocarcinoma Patients Based on Differential Scanning Calorimetry Serum Thermograms. Sci. Rep..

[40] Saito Y., Saito K., Ataké T. (1986). Theoretical Analysis of Peak Height in Classical DTA, Power-Compensated DSC and Heat-Flux DSC. Thermochim. Acta.

[41] Lim S.A.H., Antony J., Albliwi S. (2014). Statistical Process Control (SPC) in the Food Industry—A Systematic Review and Future Research Agenda. Trends Food Sci. Technol..

[42] Pudjihardjo I., Mulyana I.J., Asrini L.J. (2019). Autocorrelated Multivariate Control Chart in Cooking Oil Industry. Proc. Int. Conf. Ind. Eng. Oper. Manag..

