# Supplementary material for: DSC Phase Transition Profiles Analyzed by Control Charts to Determine Markers for the Authenticity and Deterioration of Flaxseed Oil during Storage
Source: Foods. 2023 Aug 4;12(15):2954. doi: 10.3390/foods12152954 (PMC10418391; doi:10.3390/foods12152954)
Supplement: Supplementary file 1 [file foods-12-02954-s001.zip › foods-2526962-supplementary.pdf]

Supplementary materials for

DSC phase transition profiles analyzed by control charts to determine markers for the authenticity and deterioration of flaxseed oil during storage

Mahbuba Islam [1], Anna Kaczmarek [1], Anna Grygier [2] and Jolanta Tomaszewska-Gras [1,*]

[1] Department of Food Quality and Safety Management, Poznań University of Life Sciences, ul. Wojska Polskiego 31/33, 60-624 Poznań, Poland; mahbuba.islam@up.poznan.pl (M.I.); anna.kaczmarek@up.poznan.pl (A.K.)
[2] Department of Food Technology of Plant Origin, Poznań University of Life Sciences, ul. Wojska Polskiego 31/33, 60–624 Poznań, Poland; anna.grygier@up.poznan.pl
* Correspondence: jolanta.tomaszewska-gras@up.poznan.pl

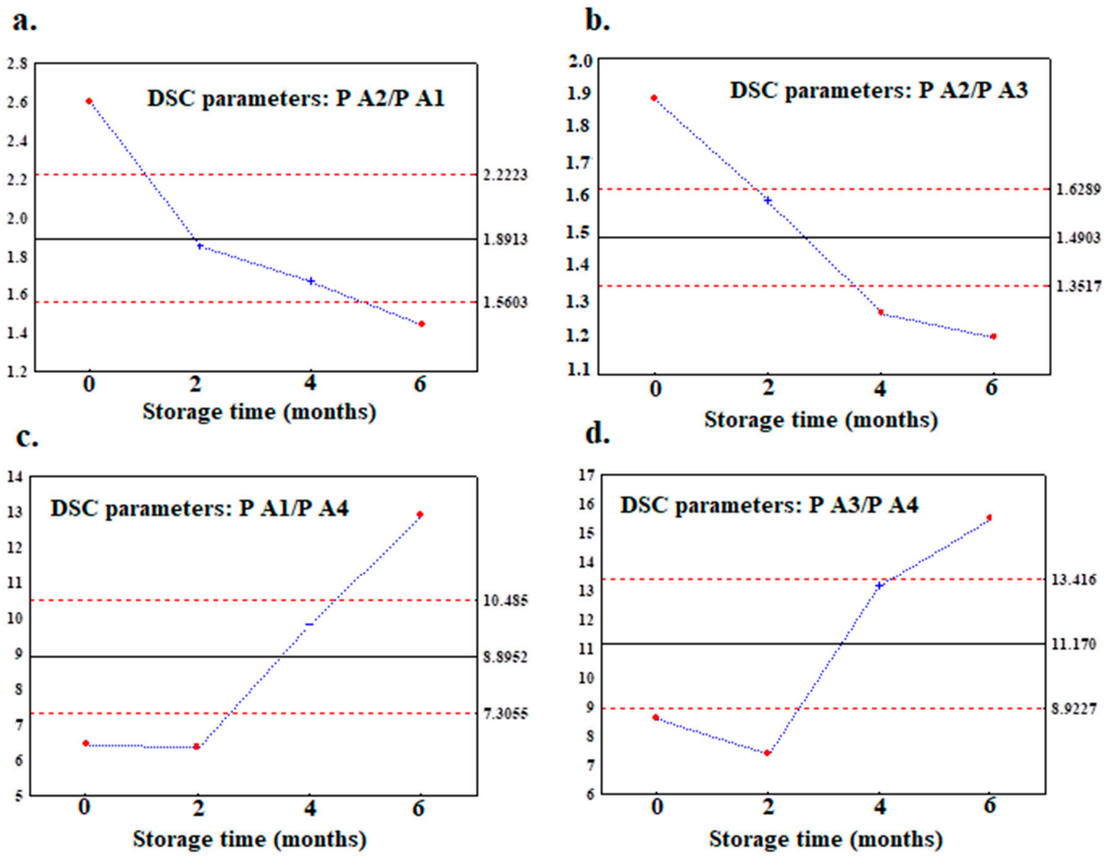

Figure S1. Control charts of unstable parameters (calculated from the ratio of peak's percentage of area) from DSC melting curves. (a) ratio of parameters P A2/P A1, (b) ratio of parameters P A2/P A3, (c) ratio of parameters P A1/P A4, (d) ratio of parameters P A3/P A4.
